# The Relationship Between Cognitive Abilities and the Decision-Making Process: The Moderating Role of Self-Relevance

**DOI:** 10.3389/fpsyg.2019.01892

**Published:** 2019-08-14

**Authors:** Menghan Jin, Lingling Ji, Huamao Peng

**Affiliations:** ^1^Institute of Developmental Psychology, Beijing Normal University, Beijing, China; ^2^Beijing Key Laboratory of Applied Experimental Psychology, Beijing Normal University, Beijing, China; ^3^Faculty of Education, Beijing City University, Beijing, China

**Keywords:** self-relevance task, decision-making process, age differences, cognitive abilities, processing speed, verbal fluency

## Abstract

This study investigated the relationship between cognitive abilities and age differences in information search and the moderating role of task self-relevance by measuring the decision-making processes of participants in both high and low self-relevance decision-making tasks. The sample included 57 young and 65 older adults. They viewed five-alternative × five-attribute decision matrices that required them to open, with a mouse click, the information cells that interested them. Processing speed, verbal fluency, working memory, and vocabulary were measured as cognitive abilities. The dependent variables were search engagement (including time-related engagement and frequency-related engagement) and search pattern (calculated based on alternative-based or attribute-based search). The results from structured equation modeling showed that age negatively predicted these cognitive abilities (processing speed, verbal fluency, working memory, and vocabulary) and positively predicted information search engagement. Processing speed mediated the effect of age on study time per cell under tasks with both high and low self-relevance. Verbal fluency, meanwhile, mediated the total search time and checking time per cell when the task was highly self-related but not when the task had low self-relevance. These results suggest that self-relevance can moderate the mediation effect of verbal fluency on the relationship between age and information search time; this means that older adults whose verbal fluency was limited require relatively more time to search information to make an informed decision. However, this effect is only sufficient when the decision-making task is highly self-related and provokes more engagement motivation toward it.

## Introduction

### Decision Making and Aging

Decision making refers to the process of selecting one option among two or more competing alternatives (Peters et al., 2011). Decision making encompasses numerous processes, including how people search for information, compare between alternatives, and select an option, as well as the outcome of the choice. The decision-making process is influenced by the characteristics of the situation and the individual decision maker (Peters et al., 2011).

Numerous studies on aging and decision making suggested there are significant differences between younger and older adults in decision-making behavior: older people search for less information than young people (Johnson, 1990; Riggle and Johnson, 1996; Mather, 2006; Yoon et al., 2009) and utilize relatively simpler search strategies (Lambert-Pandraud et al., 2005; Mata and Nunes, 2010).

Finucane et al. (2005) suggested that age differences in decision making should be interpreted in the context of a person-task fit framework to account for differences in individual and decision-making task characteristics. This is arguably a more appropriate way to understand age-related differences in decision making. It allows us to explore how developmental changes in individual variables influence the decision-making process with increasing age and over different decision-making tasks.

The primary focus of this study was the relationship between cognitive abilities and age-related differences in the decision-making process.

### Cognitive Abilities and Age Differences in Decision Making

Researchers in the field of aging have confirmed that several cognitive abilities – including working memory, processing speed, and executive control – decline with increasing age, starting in the early 20 s (Salthouse, 1991, 2004; Park et al., 2002; Wilson et al., 2002), and may negatively affect several processes implicated in decision-making.

Elderly adults’ limited cognitive abilities may negatively affect the quality or competence of their decisions. For example, Del Missier et al. (2012) explored the connection between decision-making competence, executive functioning, and general cognitive ability. They found that various measures of decision-making performance were positively associated with executive function (especially cognitive control functions), numeracy, and fluid intelligence. Furthermore, cognitive abilities were found to explain age-related differences in decision-making performance. In another study, negative relationships between age and performance were found in decision-making tasks that involved resistance to framing and applying decision rules; these relationships were mediated by cognitive abilities (Bruine de Bruin et al., 2007). Similarly, age-related decline in applying decision rules was mediated by working memory and verbal fluency (Rosi et al., 2017). Henninger et al. (2010) also found that underlying cognitive abilities (working memory and processing speed) mediated age-related differences in decision quality and the associated changes in risk preferences.

Cognitive abilities have also been associated with specific decision-making processes in older adults, such as information search and strategy use. Mata et al. (2007) asked participants to select the most expensive diamond based on clues and assessed the relationship between cognitive ability and decision-making strategies. They found that fluid intelligence and reasoning abilities specifically accounted for the age-related variance in strategy selection during the decision-making process. In other words, older adults’ age-related decline in fluid intelligence constrained their cognitive resources, leading to the use of simpler decision strategies (e.g., the *take-the-best* heuristic; Gigerenzer and Goldstein, 1996) that rely on less information. Mata and Nunes (2010) also found that older adults use more non-compensatory choice strategies, i.e., decisions typically based on less information about the potential options and fewer comparisons between options. These strategies are more straightforward and relatively simpler, and they can reduce cognitive load. Therefore, older people’s limited working memory and processing ability may cause them more difficulty when faced with decision-making tasks involving numerous alternatives (John and Cole, 1986). A meta-analysis of age-related differences in information search has also suggested that age differences in information search would likely be emphasized when more available cues are present, as this would require integrating larger amounts of information to make decisions and exert higher demands on cognitive resources (Mata and Nunes, 2010). Thus, older adults’ limited fluid cognitive abilities not only negatively affect their decision quality but are also implicated in higher cognitive load in conditions of complex and high-demand decision-making tasks. This, in turn, results in poor performance when searching for and processing available information and a reliance on less cognitively demanding strategies during decision making.

In sum, many researchers have investigated the relationship between cognitive ability and age-related differences in decision making, with a particular focus on decision-making performance or decision quality other than the decision-making process (Johnson, 1990, 1993, 1997; Johnson and Drungle, 2000; Stephens and Johnson, 2000; Mather et al., 2005; Löckenhoff and Carstensen, 2007, 2008; Ma et al., 2007; Mata et al., 2007; Reed et al., 2013; Geiger, 2017). The role of cognitive ability has repeatedly been shown to explain age differences in decision quality and strategy use. However, even when researchers investigate the decision-making process, they typically fail to examine the decision-making behavior of participants during the decision-making process itself. Information search behavior during decision-making underlies decision quality and could reveal a more detailed understanding of decision making. Information search behavior includes information engagement and search pattern, which could reveal the exact time individuals spend on each piece of information, the amount of information people process, and the pattern and organization of their search behavior (Mata and Nunes, 2010; Hess et al., 2012). This information reveals the specific effort and engagement people exert during decision making, which can lead to a better understanding of how different age groups vary in their efforts and method of processing task information, and how other factors may affect effort and engagement during the process.

### Self-Relevance and the Selective Engagement Hypothesis

Prior research has also failed to examine other factors that influence decision making – namely, engagement motivation for tasks (Bruine de Bruin et al., 2015), which may be affected by the personal relevance of the tasks (Löckenhoff and Carstensen, 2007; Hess et al., 2012). These studies showed that specific cognitive abilities can mediate decision-making performance to some extent. However, the relationship varied as a function of the research tasks and context (Bruine de Bruin et al., 2007, 2015). Thus, we speculate that the role of cognitive ability in age-related differences in decision-making performance may be influenced by aspects of the decision-making tasks, particularly the self-relevance attributed to those tasks.

Self-relevance refers to the degree of relevance between oneself and the task and could affect an individual’s motivation during the task (Hess et al., 2009a). Motivation in decision making refers to the degree of willingness to engage in decision making, which includes considering the presented information and weighing between alternatives to select the best option (Strough et al., 2015). Higher motivation during a task typically inspires more engaged effort and improved performance since one tends to be more willing to competently complete the task. Indeed, Löckenhoff and Carstensen (2007) found that age differences in performance disappeared once motivation to perform the decision-making task increased. Older adults typically perform worse than young adults when both groups are less motivated. This suggests that it is important to take motivation into account when reflecting on age differences in decision making. The effect of self-relevance is predominantly exerted through motivation, such that higher self-related tasks entail increased interest and importance and lead to increased intention and willingness to perform those tasks. Hess et al. (2009a) examined the impact of the self-relevance of decision-making tasks on the decision behavior of younger and older adults and found that age-related differences in decision performance decreased when participants reported high interest in decision-making tasks. Self-relevance also improves older adults’ memory performance regarding the task materials. Tomaszczyk et al. (2008) found that age differences in memory were reduced in pictures that were highly relevant to participants. In addition, Hess (2014) found that the self-relevance of decision-making tasks could improve older adults’ decision-making efficiency but had relatively little effect on young adults. Therefore, older adults might perform disproportionately worse than young adults in low self-related tasks due to the relatively deficient cognitive resources of older adults. Meanwhile, higher self-relevance may compensate for and enhance the performance of older adults more markedly than young adults.

Self-relevance, therefore, may play an essential role in the decision-making behavior of older adults, particularly through its effect on engagement motivation. Older adults may not perform worse on some decision-making tasks because they possess limited cognitive capacities but because they are not motivated to engage with the tasks. Yet, we will still not grasp the precise role cognitive ability plays in these tasks if we only focus on the impact of motivation. Therefore, from another perspective, if we specifically test the effect of motivation on decision making in older adults using precisely varying levels of cognitive ability, we can better understand the different mechanisms underlying cognitive ability and motivation and the relationship between them. For example, older adults may overall perform inadequately in tasks with low self-relevance as a consequence of lower intention, such that even higher cognitive abilities may have no chance of presenting competently. However, tasks with high self-relevance may arouse older adults’ involvement intentions, which would enable cognitive ability to play a relatively more important role in their decision making. In addition, the effect of self-relevance may vary according to cognitive ability, as each ability may play a different role during decision making. This research aimed to examine the role of self-relevance for older adults in the decision-making process. However, unlike previous research, which principally focused on the relationship between self-relevance and age-related differences in decision making (e.g., Meyer et al., 2007; Hess et al., 2009a, b, 2012; Hess, 2014), we sought to examine the role of cognitive abilities while taking self-relevance into account, as it is rarely discussed in the literature. Furthermore, as the prior literature has indicated that different cognitive abilities play different roles in the decision-making processes of younger and older adults, this study examined whether these relationships would vary as a function of differing levels of self-relevancy in decision-making tasks. This study simultaneously focused on two crucial age-related differences in the decision-making process: age-related changes in cognitive abilities, which may affect decision making in different degrees, and self-relevance, which may highlight the influence of cognitive abilities in older adults.

### Current Study

In the present study, a decision-making process tracing method was used to examine the relationship between various cognitive abilities and age-related differences in the decision-making process, taking the self-relevance of the decision-making task into account.

The purpose of the present study was to specify the role of cognitive ability in the decision-making process. Specifically, we posited that cognitive abilities such as processing speed would exert greater influence on the time spent on decision making because processing speed affects how quickly one can process the descriptive information in each cell and compare the information with each option. In addition, working memory may be associated with the amount of information retained during the task, as information in the former cell would be covered when viewing information in another cell; thus, limited memory capacity might lead to repeated reviewing behavior as one may easily forget. We speculate that working memory might be associated with repeated viewing, representing frequency-related information search behavior. These are essential information search index behaviors of search engagement derived from the experimental design, and copious research has confirmed the remarkable age-related characteristics of these two cognitive abilities (Hofer et al., 2003).

This study’s experimental interface contained abundant descriptive verbal information associated with task performance; thus, we also tested verbal fluency, which has been related to the use of decision-making rules (Rosi et al., 2017). The test of verbal fluency was conducted under time constraints so it could also measure the pace at which participants extracted information and made decisions. Accordingly, we hypothesized that verbal fluency might affect time-related information search behavior. In addition, vocabulary comprehension (i.e., the ability to understand descriptive information in tasks) might also affect the speed of information comprehension. A better understanding might lead to more efficient processing and comparisons, which in turn might potentially impact time-related information search behavior.

We were also interested in examining the impact of self-relevance on decision making and whether self-relevance influences the role of cognitive abilities in decision making. According to the selective engagement hypothesis, older adults are expected to engage more in tasks with high self-relevance. Thus, participants with superior cognitive abilities overall might also perform better on tasks with high self-relevance compared to tasks with low self-relevance. We posit that cognitive abilities will exert more of an effect in the high self-relevance condition.

As such, the present study posited the following hypotheses:

*Hypothesis 1*: Time-related information search behaviors

Processing speed, verbal fluency, and vocabulary comprehension will mediate the relationship between age and the duration participants expend on decision making.

*Hypothesis 2*: Frequency-related information search behaviors

Working memory will mediate the relationship between age and the frequency of information search.

*Hypothesis 3*: Information search patterns

Processing speed and working memory will mediate the relationship between age and search patterns.

*Hypothesis 4*: The effect of self-relevance

The mediating effect of cognitive ability will be stronger in tasks with high self-relevance relative to tasks with low self-relevance.

## Materials and Methods

### Participants

The sample consisted of 57 young college students (mean age *M* = 22.07, *SD* = 2.47; 19 men and 38 women) recruited from Beijing Normal University and 64 elderly adults (mean age *M* = 64.48, *SD* = 4.56; 23 men and 41 women) recruited from nearby neighborhoods. Participants received informed consent forms, demographic questionnaires, and a battery of cognitive tests and decision-making tasks for the experiment. This study was carried out in accordance with the recommendations of the Ethics Committee of the School of Psychology, Beijing Normal University, and the study was approved by this Ethics Committee. Written informed consent was obtained from all participants before the experiment.

### Decision-Making Tasks

**FIGURE 1:**
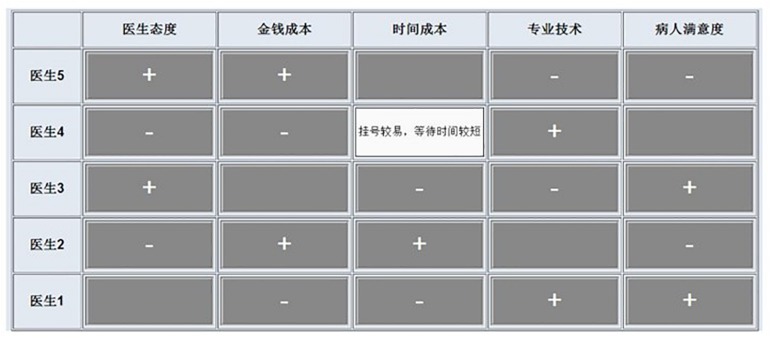
The matrix of one decision-making task. Five alternatives were presented in the rows, and five attributes were presented in the columns.

The decision-making tasks were presented using a computer screen with a mouse-tracing interface (Mouselab; Willemsen and Johnson, 2011). Mouselab Web Designer 1.00 was used to compile the experimental program interface and trace participants’ information search behavior. The computer screen presented each decision-making task on a five-alternative × five-attribute matrix. The matrix of one decision-making task is shown in Figure 1, which contains five alternatives in the row and five attributes in the column. Participants were required to search for and find descriptive verbal information by clicking on each covered information cell. The search behavior during the decision-making process was evaluated, and participants’ search engagement and search patterns were analyzed.

The decision-making tasks contained high and low self-relevance tasks. These tasks were selected using interviews and the subjective ratings of 30 young (age: *M* = 22.97, *SD* = 1.63; education years: *M* = 16.8, *SD* = 1.35) and 29 older adults (age range: *M* = 70.34, *SD* = 6.03; education years: *M* = 14.03, *SD* = 2.53). Twenty-three daily-life decision scenarios that young and older adults generally face in real life were collected through interviews about daily-life decision scenarios (e.g., “selection of physical exercise” and “selection of friends”). A survey questionnaire was constructed based on the daily decisions selected, and participants were asked to rate the importance and familiarity of each decision scenario on a seven-point Likert scale (from 1 = Not important/familiar at all to 7 = Extremely important/familiar). The participants in the rating task differed from those in the interview task. Thirty-one young (age: *M* = 20.77, *SD* = 2.23; education years: *M* = 14.35, *SD* = 1.20) and 35 older adults (age range: *M* = 68.91, *SD* = 5.80; education years: *M* = 10.79, *SD* = 3.53) participated in the questionnaire rating task. Thereafter, two personal relevance levels of decision-making tasks were selected based on points on the measures of importance and familiarity. These tasks were screened to ensure that no significant age differences in importance and familiarity existed in tasks with equal levels of personal relevance. Significant task differences in importance and familiarity were also present between tasks of high and low self-relevance.

High self-relevance tasks (i.e., high degree of importance and familiarity) included “selection of career plan” and “selection of friends” for young adults and “selection of doctor” and “selection of friends” for older adults. Low self-relevance tasks contained items on “purchasing car” and “selection of nursing home” for young adults and “purchasing car” and “selection of career plan” for older adults. The descriptors of importance and familiarity contained in the high and low self-relevant tasks are presented in Table 1. After the decision scenarios were determined, the five most important attributes for each decision scenario were determined through previous interview results and suggestions from experts in each domain. Each decision matrix task was presented with five alternatives and five attributes for each option. The five attributes for each alternative in the decision-making tasks were presented as two positive attributes, two negative attributes, and one neutral attribute. This ensured that there was no absolute best or worst option in each task. Participants were required to select one optimal option based on their own reasoning during each task.

**TABLE 1:** Description of importance and familiarity for the two tasks.

**Measures**	**Group**	**High self-relevance**		**Low self-relevance**	
			
		***M***	***SD***	***M***	***SD***
Importance	Young	6.01	0.92	2.30	1.30
	Old	5.79	1.03	1.93	0.61
Familiarity	Young	5.97	1.08	2.48	1.61
	Old	5.80	1.04	2.39	0.61

The dependent variables were search engagement (including time-related engagement and frequency-related engagement) and search pattern (calculated based on alternative-based or attribute-based search). These decision-making indices are explained in further detail below.

#### Search Engagement

Search engagement was viewed as the level of cognitive effort and the degree of engagement in the search behaviors implicated in the task, which were measured according to information search time and the amount of information sampled (reflected by information search frequency in present study design) (Hess et al., 2012; Hess, 2014). For the sake of clarity, we classified search engagement into two categories: time-related engagement and frequency-related engagement.

Time-related engagement included (a) checking time per cell: the mean checking time spent on searching an opened cell in one decision-making task, calculated based on decision-making time/total number of views; and (b) decision-making time: the total decision time in one decision-making task, calculated based on the time interval from the participants’ starting click to their submission of a choice in the decision-making task.

Frequency-related engagement included (c) the frequency of views per cell: the mean number of views for one opened information cell in a particular decision-making task, calculated based on the total number of views/search breadth; (d) the number of repeated views: the total number of boxes that were repeatedly searched in the decision-making task; (e) the frequency of total views: the total number of views of all opened information cells in a particular decision-making task; and (f) search breadth: the total number of the boxes opened at least once in a decision-making task.

#### Search Pattern

Search pattern (g) was defined as the relative use of alternative-based versus attribute-based processing and reflected the general tendency of search behavior while clicking open information cells (Payne et al., 1988). Search pattern was calculated based on the Payne index. The operational definition of search pattern in the Payne index is that search pattern score = (the number of alternative-based transitions – the number of attribute-based transitions)/(the number of alternative-based transitions + the number of attribute-based transitions); the scores range from −1.0 to + 1.0 (Payne, 1976). A positive score indicates a relative tendency to search for information based on alternatives while a negative score indicates a relative tendency to search for information based on attributes. The pattern of alternatives-based search may be more systematic, as this strategy allows people to compare alternatives after acquiring enough attribute information for each option during the information search. Meanwhile, the pattern underlying attributes-based search might be more time-saving and efficient, as it involves comparing alternatives only based on relatively fewer alternatives of interest (Reisen et al., 2008).

### Measurements

#### Processing Speed

Processing speed was measured using letter comparison (Salthouse and Meinz, 1995). Participants were asked to make rapid judgments about whether a pair of digit strings were the same (e.g., 482–482, 65382–65382, and 658331–656331) in the given time (90 s). The processing speed score was measured by the maximum number of items one could complete correctly in 90 s. The higher the score, the higher the speed of information processing.

#### Working Memory Span

Working memory was measured using the backward digit span task from the Wechsler Adult Intelligence Scale (WAIS), 3rd edition (Wechsler, 1997). A series of digit strings were read to participants, which they were required to recite backward. Working memory span was measured by the maximum length of digit strings one could repeat correctly. The longer the length of the digit strings one could repeat correctly, the better one’s working memory ability.

#### Verbal Fluency

Verbal fluency was measured using the verbal fluency test in the WAIS (Wechsler, 1997). Participants were asked to state as many examples of a specific category (e.g., fruit, animal, and vegetable) in 60 s. The more examples they provided, the better their demonstrated verbal fluency.

#### Vocabulary Comprehension

The vocabulary scale from the WAIS (Wechsler, 1997) was used to measure participants’ verbal comprehension ability. The formal test comprised 10 Chinese word items that required participants to explain the meaning of every word. Every item was scored (0–2 points) according to the WAIS scoring standard, and the total score varied from 0 to 20 points. The higher the total score, the better one’s vocabulary comprehension ability.

### Procedure

Participants in two age groups were asked to complete decision-making tasks of both high self-relevance and low self-relevance.

First, participants filled out demographic information, including gender, age, education, family income, career, and self-reported health (five-point scale, from 1 = extremely bad health condition to 5 = extremely good health condition). Data for family income per month (yuan) were rated on a seven-point scale, from 1 = 0–5000 yuan to 7 = 30001–35000 yuan.

Next, four decision-making tasks were presented on a computer screen in random order for each participant using the Mouselab program. Participants were provided with two practice tasks (3 × 3 matrix) prior to commencing the formal experimental tasks. Each experimental task was presented in a 5 × 5 matrix, requiring participants to click on a cell to reveal the information inside that cell. At the end of each task, participants rated their motivation, decision experience, and the importance and familiarity of the decision. These decision-making-relevant indices are explained below.

Decision-making motivation: Participants rated the extent of their motivation to engage while making decisions in each task on a seven-point scale (from 1 = extremely low to 7 = extremely high).

Decision-making experience: Participants rated their experience of each decision-making task on a five-point scale (from 0 = no similar experience at all to 4 = four times or more).

Importance and familiarity: Participants also rated the importance and familiarity of the decision on a seven-point scale (from 1 = not important or familiar at all to 7 = very important or familiar) after every decision.

Finally, after completing the decision-making tasks, participants were asked to finish a series of cognitive tests assessing their processing speed, working memory span, verbal fluency, and vocabulary comprehension. Each participant received ¥80 in cash for participating in the study.

## Results

### Age-Related Characteristics on Demographic Variables and Cognitive Abilities

Table 2 presents the descriptive and *t*-test results for the demographic variables and cognitive abilities of the two age groups.

**TABLE 2:** Descriptive statistics and *t*-test results for both age groups.

	**Young adults**		**Older adults**		**T *df* = 119**
	**(*N = 57*)**		**(*N = 64*)**		
			
**Measures**	***M***	***SD***	***M***	***SD***	
Age (years)	22.07	2.47	64.48	4.56	−64.50^∗^
Education (years)	15.53	1.91	11.76	2.54	9.14^∗^
Family income	2.44	1.46	1.94	0.75	2.32^∗^
Self-reported health	3.84	0.56	3.14	0.77	5.65^∗∗^
Processing speed	30.16	6.42	15.81	4.26	14.29^∗^
Working memory	6.95	1.81	5.05	1.45	6.33^∗^
Verbal fluency	57.04	8.98	51.95	9.79	2.96^∗^
Vocabulary comprehension	15.79	2.33	14.28	2.88	3.14^∗^

*^∗^Correlation is significant at the 0.05 level; ^∗∗^Correlation is significant at the 0.01 level; self-report health condition was tested on a five-point scale, from 1 = extremely bad health condition to 5 = extremely good health condition). Data of family income per month (yuan) were transformed into a seven-point scale, from 1 = 0–5000 yuan to 7 = 30001–35000 yuan).*

Age differences in these variables were mostly consistent with the previous literature. Young adults reported significantly higher education years, family monthly income, self-reported health condition, processing speed, verbal fluency, and working memory span.

The only inconsistency with the literature had to do with the younger adults’ better vocabulary comprehension in the present study; this contradicts some prior literature that indicated that crystallized intelligence, such as vocabulary comprehension, usually improves over time (Schaie, 1996; Salthouse, 2004). This inconsistency could be attributable to differences in the amount of education between the two age groups. This will be discussed and analyzed in the section “Discussion”.

### Manipulation Check for Self-Relevance

The Statistical Package for Social Sciences 22.0 was used to check the self-relevance manipulation in our experimental tasks by examining differences in the self-reported ratings of importance, familiarity, and motivation in each task during the two conditions of self-relevance, using a repeated-measures ANOVA.

As we can see in Table 3, the manipulation of self-relevance was significant in both the young and older groups under the indices of importance, familiarity, and motivation. These results confirmed that the manipulation of self-relevance in the current decision-making tasks was successful.

**TABLE 3:** Descriptive and *t*-test results of subjective evaluation of importance, familiarity, and motivation of decision-making tasks.

		**High**		**Low**			
**Measures**	**Group**	**self-relevance**		**self-relevance**		**df**	**t**
					
		***M***	***SD***	***M***	***SD***		
Importance	Young	5.92	0.85	2.39	1.09	56	22.70^∗∗^
	Old	5.56	1.01	3.00	1.38	63	12.97^∗∗^
Familiarity	Young	5.09	0.86	3.04	1.12	56	10.97^∗∗^
	Old	5.17	1.21	3.61	1.37	63	6.79^∗∗^
Motivation	Young	5.43	0.90	3.85	1.39	56	8.92^∗∗^
	old	4.87	1.07	3.98	1.23	63	5.32^∗∗^

*^∗^F is significant at the 0.05 level; ^∗∗^F is significant at the 0.01 level.*

### Role of Cognitive Abilities in Age and Decision-Making Performance

#### Age, Cognitive Abilities, and Information Search Performance

The descriptive statistics for information search behaviors are presented in Table 4. First, we examined the correlations between cognitive abilities and information search indices to form a preliminary understanding of the relationship between age, cognitive abilities, and information search behavior. As shown in Table 5, which presents the results for the correlations between age, cognitive abilities, and information search indices, age was significantly negatively related to several cognitive abilities (processing speed, working memory, verbal fluency, and verbal comprehension) and generally significantly related to most of the search engagement indices and subjective decision-making evaluations in both the high and low self-relevance conditions. Meanwhile, these four cognitive abilities had significantly negative relationships with several search engagement indices. However, the search patterns in both conditions of self-relevance only showed non-significant correlations with age and the various cognitive abilities.

**TABLE 4:** Descriptive statistics of decision-making process indices in older and young adults.

**Measures**	**High self-relevance**		**Low self-relevance**	
		
	**Young M *(SD)***	**Old M *(SD)***	**Young M *(SD)***	**Old M *(SD)***
**Time-related engagement**				
Checking time per cell(s)	1.26 (0.56)	2.90 (1.55)	1.50 (0.94)	4.10 (5.56)
Decision-making time(s)	19.75 (13.54)	56.30 (37.69)	17.07 (11.40)	56.60 (37.53)
**Frequency-related engagement**				
Views per cell	0.65 (0.43)	0.87 (0.57)	0.51 (0.34)	0.70 (0.45)
Repeated viewings	3.18 (3.23)	4.52 (4.67)	2.21 (2.32)	3.61 (3.78)
Total views	16.10 (11.02)	21.09 (14.25)	12.60 (8.45)	17.76 (11.30)
Search breadth	11.84 (6.32)	15.40 (7.08)	10.53 (5.67)	13.15 (7.38)
Search pattern	0.37 (0.48)	0.52 (0.48)	0.35 (0.47)	0.38 (0.53)

*Checking time per cell: the mean search time spent studying a cell. Decision-making time: the total time spent on search in one task. Views per cell: the mean views opening the same cell in one task. Repeated viewings: the number of box searched repeatedly. Total views: the total views for all opened cells of one task. Search breadth: the total number of cells searched at least once. Search pattern: positive values indicate a more alternative-based search and negative values indicate a more attribute-based search.*

**TABLE 5:** Pearson’s correlation results between age, cognitive abilities, and decision-making performance.

	**Age**	**PS**	**WM**	**VF**	**VC**
Age	1	–0.84^∗∗^	–0.49^∗∗^	–0.28^∗∗^	–0.25^∗∗^
**H-Time-related engagement**					
H-time per cell	0.60^∗∗^	–0.57^∗∗^	–0.37^∗∗^	–0.37^∗∗^	−0.22^∗^
H- Decision-making time	0.55^∗∗^	–0.48^∗∗^	–0.35^∗∗^	–0.27^∗∗^	–0.06
**H-Frequency-related engagement**					
H-views per cell	0.22^∗^	–0.15	–0.11	–0.04	0.06
H-repeated viewing	0.18	–0.12	–0.05	–0.042	0.049
H-total views	0.23^∗^	–0.16	–0.11	–0.06	0.06
H-search breadth	0.26^∗∗^	–0.14	–0.09	0.017	0.02
H-search pattern	0.12	–0.08	–0.06	–0.11	0.05
**L-time-related engagement**					
L-time per cell	0.32^∗∗^	–0.29^∗∗^	–0.27^∗∗^	–0.15	–0.03
L- decision-making time	0.58^∗∗^	–0.50^∗∗^	–0.33^∗∗^	–0.27^∗∗^	–0.14
**L-frequency-related engagement**					
L-views per cell	0.24^∗∗^	−0.19^∗^	–0.07	–0.07	–0.05
L-repeated viewing	0.22^∗^	−0.20^∗^	–0.11	–0.09	–0.01
L-total views	0.25^∗∗^	−0.19^∗^	–0.07	–0.09	–0.04
L-search breadth	0.19^∗^	–0.12	0.00	–0.01	–0.03
L-search pattern	0.01	–0.02	–0.05	0.04	–0.15

*^∗^Correlation is significant at the 0.05 level; ^∗∗^correlation is significant at the 0.01 level. PS, processing speed; WM, working memory; VF, verbal fluency; VC, vocabulary comprehension; H- means decision-making index under high self-relevance tasks and L- means low self-relevance tasks.*

We further explored the exact effects of specific cognitive abilities and sought to verify the possible mediation effects of cognitive abilities on the relationship between age and information search behavior during decision making.

#### Mediating Effect of Cognitive Ability on Time-Related Information Search Behavior

The structural equation model for age, cognitive abilities, and information search performance was created in Amos 21.0 (Arbuckle, 2006) for the present analysis. Specifically, a bootstrapping approach was used to examine the mediating effects of the various cognitive abilities, which proved to have greater statistical power than the Baron and Kenny (1986) and Sobel (1986) method (Shrout and Bolger, 2002; MacKinnon et al., 2004; Preacher and Hayes, 2008; Hayes, 2009; Bolin, 2014). The analysis under this approach randomly selected 5000 bootstrapped samples from the dataset to compute bias-corrected confidence intervals to check the mediation effect (the widest biased-corrected confidence interval should not include zero) under a confidence interval of 95%. Using a method similar to that of Rosi et al. (2017), we first conducted single mediation analyses on each cognitive ability and the information search indices to find out which cognitive ability mediated age and information search indices. Subsequently, multiple mediation analyses were conducted combining the cognitive abilities that were significant mediators in single mediation analysis to generate more comprehensive results (Rosi et al., 2017).

The results of the single mediation analysis showed that only verbal fluency and processing speed exhibited a significant mediation effect between age and time-related information search behavior. The remaining two cognitive abilities (working memory and verbal comprehension) had non-significant mediation effects. In addition, no evidence was found for hypotheses 2 and 3, which predicted the relationship of cognitive abilities with frequency-related engagement and search pattern.

Specifically, verbal fluency showed a significant mediation effect on mean checking time per cell (indirect effect = 0.005, *p* < 0.01) and total decision time (indirect effect = 0.072, *p* < 0.05) as dependent variables in the high self-relevance condition after controlling for demographic variables (gender, family income, and education). Processing speed also showed strong mediation effects when using mean checking time of both high and low self-relevance condition as dependent variables (indirect effect = 0.013, *p* < 0.01 for high self-relevance; indirect effect = 0.02, *p* < 0.05 for low self-relevance). These results confirm parts of hypothesis 1 – namely, that processing speed and verbal fluency mediated the relationship between age and the time participants spent searching for information.

**FIGURE 2:**
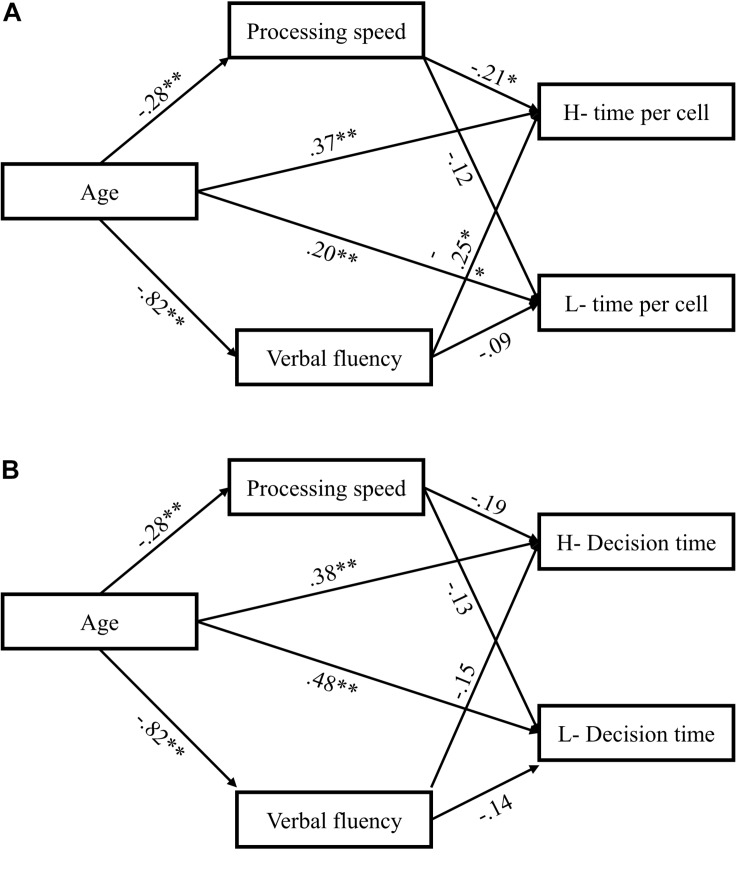
Graphic representations of the multiple-mediation model across potential mediators (processing speed and verbal fluency) were significant in the single-mediation models and standard regression weightings of each relationship. **(A)** The multiple-mediation model across potential mediators on mean checking time per cell; **(B)** the multiple-mediation model across potential mediators on decision time. ^∗^Regression is significant at the 0.05 level; ^∗∗^regression is significant at the 0.01 level. H- means decision-making index under high self-relevance tasks, and L- means low self-relevance tasks.

We then combined processing speed and verbal fluency in our final model as two mediators to reconfirm the respective effects of each mediator in a more comprehensive model, still using mean checking time per cell and total decision time in both self-relevance conditions as four dependent variables while controlling for demographic variables (gender, family income, and education). The final model fit reasonably well (CFI = 0.959; RMSEA = 0.085). The multiple mediation model and specific regression weights are presented in Figure 2. We examined the A × B indirect effect of each mediator individually (path A represents relationship from age to the mediator; path B represents the relationship from the mediator to the dependent variable) (Hayes, 2009; Bolin, 2014). Table 6 shows the indirect effects of each mediator in the model and the 95% confidence intervals of the indirect effect estimates. Table 7 reports the total indirect effect, total direct effect, total effect of age on decision time, and mean checking time in the model. As we can see from the results, there was a mediation effect of processing speed on mean checking time per cell in both conditions of self-relevance (indirect effect = 0.012, *p* < 0.05 for high self-relevance; indirect effect = 0.02, *p* < 0.05 for low self-relevance). Verbal fluency had a significant mediating effect on mean checking time per cell (indirect effect = 0.05, *p* < 0.01) and total decision time (indirect effect = 0.07, *p* < 0.05) but only in the high self-relevance condition.

**TABLE 6:** The effect of mediators in the multiple mediation model.

**Decision making**	**Indirect effect (Bootstrap 95% CI)**	
	
**Process**	**Verbal fluency**	**Processing speed**
H-time per cell	0.005^∗∗^(0.001, 0.014)	0.012^∗∗^(0.002, 0.023)
L-time per cell	0.005 (0, 0.019)	0.020^∗∗^(0.02, 0.057)
H-decision time	0.070^∗^(0.003, 0.236)	0.249 (−0.014, 0.648)
L-decision time	0.063 (−0.009, 0.211)	0.173 (−0.113, 0.471)

*^∗^Mediation is significant at the 0.05 level; ^∗∗^mediation is significant at the 0.01 level. H-means decision-making index under high self-relevance tasks and L- means low self-relevance tasks.*

**TABLE 7:** The standardized indirect, direct effect, and total effect of age on decision-making process in a multiple mediation model.

**Decision making**	**Total Indirect effect of age**	**Direct effect of age**	**Total effect of age**
Process			
H-time per cell	0.196^∗∗^	0.376^∗∗^	0.573^∗∗^
L-time per cell	0.145	0.477^∗∗^	0.622^∗∗^
H-decision time	0.243^∗∗^	0.373^∗∗^	0.616^∗∗^
L-decision time	0.124	0.197^∗∗^	0.321^∗∗^

*^∗^Mediation is significant at the 0.05 level; ^∗∗^mediation is significant at the 0.01 level. H-means decision-making index under high self-relevance tasks and L- means low self-relevance tasks.*

#### Moderating Role of Self-Relevance in the Mediating Effect of Cognitive Ability

We examined the moderating effect of self-relevance on the tasks with the mediating effects of cognitive ability. We already separated the dependent variables into decision-making performance in the high self-relevance condition and performance in the low self-relevance condition and examined the differences in mediation effects under these two conditions.

As can be seen from the mediation results described above, verbal fluency had a significant mediation effect on time-related information behavior only in high self-relevance conditions, and the mediation effect disappeared when self-relevance was low, suggesting that self-relevance moderated the mediation effect of verbal fluency. These results confirm hypothesis 4, which predicted the moderating role of self-relevance. However, the mediation results for processing speed were significant in both the high and low self-relevance conditions. As such, it appears that self-relevance had no moderating effect on the mediation of processing speed.

## Discussion

The present study used a process-tracking procedure to examine the relationship between cognitive abilities and age-related differences in information search during decision making. The mediation analysis showed that only processing speed and verbal fluency exerted significant mediation effects on the age and information search indices. Specifically, the mediation effects of processing speed on the mean checking time per cell were present in both high and low self-relevance tasks, whereas verbal fluency only showed a mediation effect on the mean checking time and total decision time in the high self-relevance condition. This suggests a moderating effect in self-relevance: when the decision-making tasks are highly self-relevant, older adults did significantly better in mobilizing their verbal fluency for more efficient information searches.

### Age-Related Characteristics in the Decision-Making Process

The results of the present study showed that searching among large swathes of information and expending substantial time and energy in decision-making tasks were distinctive features in older adults’ decision-making processes relative to those of young adults.

Search engagement was found to have significant relationships with age, including time-related engagement and frequency-related engagement, as shown in correlation results in Table 5. These results might be related to older adults’ diminished cognitive abilities, which resulted in their relatively reduced processing speed and ability to deal with cognitively demanding decision-making tasks. Specifically, older adults demonstrated significantly lower processing speed levels; thus, they took longer to open information cells and compare between alternatives. Limited working memory also led them to click open cells and compare information repeatedly more often.

No relationship between age and search pattern was found. Similarly, in both Hess et al. (2012) and Queen et al. (2012), no significant age differences were found in the search organization, which is comparable to the search pattern in this study. Moreover, the information matrices presented in our tasks were less complex and elaborate than those in the aforementioned studies, which might have made them easier to grasp, thus producing less searching behavior. Therefore, taking these factors into account, the non-significant relationship between age and search pattern was not a surprising outcome.

### Relationship Between Cognitive Abilities and Age-Related Differences in Decision Making

#### Mediating Effect of Verbal Fluency and the Role of Self-Relevance

Verbal fluency was found to exert a mediating effect on time-related indices only in tasks with high self-relevance. As hypothesized, older adults had more motivation to perform better in high self-relevance decision-making tasks. Thus, older adults’ limited verbal fluency might hamper their speed in comprehending verbal information from each cell and the total of decision-making tasks. It would therefore take older adults longer to discover and comprehend information if they wanted to increase their performance in these tasks. On the contrary, older adults reported relatively low motivation to systematically search for and compare information in low self-relevance tasks. Lack of verbal fluency did not significantly influence age-related differences in search time. The results from the correlations were also consistent with our inference that verbal fluency showed a significant negative correlation with total decision time and mean checking time in the high self-relevance condition; yet, the correlation was no longer significant in the low self-relevance condition. Moreover, when we compared the total effect of age on decision making in the high and low self-relevance conditions, as shown in Table 7, the total effect of age was lower in the high self-relevance condition relative to the low self-relevance condition for mean search time per cell (0.573 vs. 0.622). However, it was higher in the high self-relevance condition relative to the low self-relevance condition for the total decision time (0.616 vs. 0.321). Thus, when self-relevance was high, older adults felt more motivated to engage in the task and thus processed information more efficiently and required less mean checking time. The effect of task relevance on older adults might be more compensatory relative to young adults, which led to a lower age effect on mean checking time in the condition of high self-relevance. However, we found a reverse result for total decision time. Total decision time resulted from the mean checking time multiplied by the total views in each task; thus, total decision time was, in addition, affected by search frequency. Higher self-relevance led to higher total views for both age groups and accordingly led to longer total decision time. This effect from total views might overwhelm the relative influence of individuals’ search time and finally result in a higher age effect for total decision time in the condition of higher self-relevance. The results from the repeated measures ANOVA (2 ages: old, young × 2 self-relevancies: high; low) also confirmed our speculations, as the total views in the condition of high self-relevance were significantly higher than in the condition of low self-relevance (*F* = 15.28; *P* < 0.001; η^2^ = 0.114), mean checking time in high self-relevance was only marginally significantly less than in low self-relevance (*F* = 3.57; *P* < 0.061; η^2^ = 0.029), and the main effect of self-relevance was not significant for total decision time.

In conclusion, self-relevance did have some positive influence on older adults in decision making. Older adults searched for more information in the task and processed information more efficiently through the effect of cognitive ability, like verbal fluency. Also, the effect of age on the speed of processing information would be tendered.

#### The Mediating Effect of Processing Speed

Processing speed may mediate the relationship between age and mean checking time in both high and low self-relevance tasks. This might be due to the fundamental role of processing speed in cognitive aging. Processing speed is a more basic cognitive ability that showed extremely large age-related differences, and it affects numerous aspects of cognitive performance (Anstey et al., 2003; Ghisletta and Lindenberger, 2003; Finkel et al., 2005, 2007; Finkel et al., 2009). It might be inferred that the effect of processing speed on older adults’ decision-making behavior is hardly affected by self-relevance or other contextual factors. These results suggested that the influence of the diminished processing speed characteristic in older adults might be strong enough to affect their time spent on daily decision making, regardless of its importance and familiarity.

#### Non-significant Results in Information Search Indices

These mediation effects only affected time-related indices. In this study, cognitive ability did not explain age-related differences in search breadth and search pattern. Compared to search breadth and search pattern, time-related indices had more significant and stronger correlational relationships between ages (shown in Table 4). Therefore, diminished cognitive abilities related to age could explain the many age-related differences in the time-related indices relative to other information search indices. It is possible that some task structure factors aside from age and cognitive ability have an important effect on search breadth and pattern, such as task complexity. Future research should continue to explore and examine other potential task-related factors.

#### Non-significant Results in Cognitive Abilities

The present study only found effects in verbal fluency and processing speed while the effects on working memory were too weak to reach significance. However, research on cognitive aging has found that age-related differences explained by working memory decreased substantially after controlling for processing speed (Salthouse and Meinz, 1995; Park, 2000). Thus, processing speed might exert a more intensive effect on decision making relative to working memory. As such, working memory did not show any mediation effects in the current study. Meanwhile, the task used in our study consisted of only five options and five attributes for each, while every attribute contained only three types of content, which might be understood as a relatively low memory load for participants. The abovementioned factors might account for the non-significant role of working memory in this study.

Vocabulary comprehension in the present study indicated that an age-related decreasing tendency was not consistent with some research on crystallized ability. However, some norm-revision studies of intelligence scales (e.g., Wechsler scale, Woodcock-Johnson psychological education test) also noted that vocabulary performance appears to have a tendency to decrease from age 50 onward (Salthouse, 2003). In particular, some studies conducted on a sample of older Chinese adults indicated that the vocabulary of older adults decreased significantly after a 20-month trace (Peng et al., 2009). These untraditional vocabulary trajectories might be caused by educational differences or the overwhelming effect of forgetting past knowledge on obtaining new knowledge for older adults.

Vocabulary showed no mediating role in age-related differences in decision making. This result might have been mediated by the following. First, the dependent variables in the present study focused predominantly on decision-making processes but not on decision quality. Decision-making processes involved clicking on a cell to search for information and reflecting on the available options, which might relate more directly to fluid cognitive abilities. Second, although rich experiences and domain-specific knowledge benefit older adults’ decision-making quality as they are able to utilize heuristic strategies to reduce cognitive load (Queen et al., 2012; Pachur and Marinello, 2013; Li et al., 2015), vocabulary is a domain-general crystallized ability, and the specific age pattern (decreasing with age) observed in present results hardly predicted decision-making performance. Lastly, the verbal description of the information provided in the task presentation was relatively brief and easy to understand. Therefore, the finding that vocabulary comprehension had no mediating effect in the relationship between age and decision-making processes is reasonable.

### Limitations and Future Directions

The significance of the present study lies in the fact that we examined the exact cognitive abilities that underlie, or are possibly connected to, the information search process during decision making. The results also reconfirmed the fundamentally important role of processing speed in information search. This finding is instructive and represents a meaningful contribution to furthering the development of cognitive interventions for older adults. We also found that verbal fluency had an important effect on information searching, especially in the context of high self-relevance. These results captured a semblance of the relationship between cognitive ability and self-relevance in decision making and require further study in future research. In addition, there are several limitations in our study that require further exploration and consideration in future research.

First, we only measured the indices of the decision-making process in this study and did not consider objective decision quality, which also is vital to revealing consequences and meanings after decision making. Furthermore, the relationship between cognitive ability and objective decision quality in older adults is also worthy of future investigation.

Second, this study only tested one crystallized cognitive ability – vocabulary comprehension. Future research should consider the role of crystallized abilities in decision making, especially abilities associated with domain-specific knowledge and experience. Rich experiences and domain-specific knowledge have been found to offset older adults’ decision quality (Li et al., 2015).

Third, the computerized tasks used in the present study might have generated confounding effects, as differences in computer experience between age groups, which were not controlled or measured, might have affected their performance. This problem requires more attention and better and more effective control in future research.

Lastly, the within-subject design of self-relevance in the current study might have introduced some statistical confusion. We were trying to compare the differences between the mediating effects for the high and low self-relevance conditions, and a between-subjects design is typically used for traditional grouping variables for multigroup comparison and analysis. In future research, an appropriate experimental design that is better suited for more rigorous and in-depth statistical analysis should be adopted.

## Conclusion

The present study found and confirmed the mediating effect of processing speed and verbal fluency in terms of age-related differences in the decision-making process. The role of personal relevance in decision-making tasks was also demonstrated in the moderating effects of verbal fluency.

## Ethics Statement

This study was carried out in accordance with the recommendations of the Ethics Committee of the School of Psychology, Beijing Normal University and approved by the Ethics Committee of the School of Psychology, Beijing Normal University. All subjects gave written informed consent in accordance with the Declaration of Helsinki.

## Author Contributions

MJ analyzed the data, wrote the manuscript, and helped to conduct the experiments and collect the data. HP designed the study and revised the manuscript. LJ involved in the experimental design and the data collection.

## Conflict of Interest Statement

The authors declare that the research was conducted in the absence of any commercial or financial relationships that could be construed as a potential conflict of interest.
